# Epigenetic Regulation Involving microRNAs in Diabetes

**DOI:** 10.3390/biom16050742

**Published:** 2026-05-19

**Authors:** Dmitriy Ivanov, Anna Drobintseva, Andrey Ivanov, Yulia Belova, Lilya Ditkovskaya, Olga Maryina, Igor Kvetnoy, Ruslan Nasyrov, Elena Semenova

**Affiliations:** 1 Department of Neonatology with Training in Neurology and Obstetrics and Gynecology, Faculty of Postgraduate and Additional Professional Education, Saint-Petersburg State Pediatric Medical University, Litovskaya Ulitsa, 2, 194100 St. Petersburg, Russia; doivanov@yandex.ru; 2 Department of Histology and Embryology Named After Prof. A.G. Knorre, Saint-Petersburg State Pediatric Medical University, Litovskaya Ulitsa, 2, 194100 St. Petersburg, Russia; andrey.v.ivanov@spbu.ru (A.I.); bi.day@mail.ru (Y.B.); 3 Saint-Petersburg State University Hospital, Fontanka Embankment, 154, 191023 St. Petersburg, Russia; 4 Department of Childhood Diseases Named After Prof. I.M. Vorontsov, Faculty of Postgraduate and Additional Professional Education, Saint Petersburg State Pediatric Medical University, Litovskaya Ulitsa, 2, 194100 St. Petersburg, Russia; liliya-ditkovskaya@yandex.ru (L.D.); olga210697@yandex.ru (O.M.); 5 Department of Pathological Anatomy with a Course in Forensic Medicine Named After Prof. D.D. Lokhov, Saint Petersburg State Pediatric Medical University, Litovskaya Ulitsa, 2, 194100 St. Petersburg, Russia; igor.kvetnoy@yandex.ru (I.K.); rrmd99@mail.ru (R.N.); 6 Petersburg Nuclear Physics Institute Named by B.P. Konstantinov of National Research Centre «Kurchatov Institute», Mcr. Orlova Roshcha 1, 188300 Gatchina, Russia; semenova_el.spb@mail.ru

**Keywords:** diabetes, microRNAs, autoimmune process, early diagnosis, personalized medicine

## Abstract

Diabetes mellitus (DM) is a group of metabolic diseases characterized by chronic hyperglycemia resulting from defects in insulin secretion, insulin action, or both. The most common types—type 1 and type 2 diabetes—have different etiologies and pathophysiological mechanisms. Type 1 diabetes (T1DM) results from autoimmune destruction of the insulin-producing pancreatic β-cells, leading to the development of absolute insulin deficiency, whereas in type 2 diabetes (T2DM), impaired carbohydrate metabolism is primarily caused by insulin resistance and relative insulin deficiency. Current diagnostic criteria do not allow for the detection of the disease at the preclinical stage. MicroRNA (miRNA) influences post-translational regulation of gene expression by inhibiting mRNA translation and also promotes mRNA degradation. The aim of this review is to summarize current evidence on the role of microRNAs in the pathogenesis of T1DM and T2DM and to evaluate their potential as early diagnostic biomarkers and therapeutic targets. It is demonstrated that T1DM and T2DM exhibit altered expression of specific microRNAs involved in β-cell apoptosis, autoimmune inflammation, and insulin signaling. In T1DM, key miRNAs include miR-21, miR-25, miR-146a, and miR-375, which reflect β-cell destruction and the autoimmune process. In T2DM, critical roles are played by miR-9, miR-29, miR-34a, miR-103/107, miR-126, miR-143, and miR-375, which regulate insulin secretion, lipid metabolism, and tissue insulin sensitivity. Particular attention is given to microRNAs whose expression changes several years before clinical disease onset (miR-15a, miR-126, miR-375), offering opportunities for early diagnosis. Data are presented on circulating miRNAs in stable biological fluids (blood, urine). It should be emphasized, however, that the proposed microRNA panel currently represents only a potential diagnostic tool. This panel requires further validation and confirmation by clinicians in large-scale prospective studies and does not yet claim to be ready for routine clinical use. Nevertheless, the development of such a universal microRNA panel, followed by thorough clinical evaluation, has promising biomedical potential, which will not only allow for the diagnosis of diabetes at an early stage but also identify new therapeutic targets for personalized medicine.

## 1. Introduction

Diabetes mellitus is a group of metabolic diseases characterized by chronic hyperglycemia, as a result of impaired insulin secretion, the action of insulin, or both of these factors. The most common types, type 1 diabetes mellitus (T1DM) and type 2 diabetes mellitus (T2DM), have different etiologies and pathophysiological mechanisms. T1DM occurs as a result of autoimmune destruction of insulin-producing beta cells of the pancreas, followed by the development of absolute insulin deficiency, while in T1DM, impaired carbohydrate metabolism is mainly due to insulin resistance and relative insulin deficiency.

According to the International Diabetes Federation (IDF), in 2024, diabetes affected 11.11% of the global adult population, corresponding to 589 million adults [1,2]. Projections indicate that by 2050, diabetes will affect 12.96% of adults aged 20–79 years, totaling 853 million. T1DM accounts for 10–12% of cases, T2DM for 85–90%, other types of diabetes for less than 1%, and gestational diabetes mellitus develops in approximately 2–4% of pregnant women on average. Among children and adolescents, T1DM is more common, accounting for up to 90% of all diabetes cases [3]. The increasing prevalence of diabetes poses a long-term threat and leads to a reduction in healthy life expectancy. Furthermore, its systemic microvascular complications—including nephropathy, retinopathy, and damage to the macrovessels of the heart and brain, and lower limb arterial disease—are frequent causes of disability and mortality among patients with diabetes [3].

Predisposition to the development of autoimmune diabetes mellitus is determined by multiple genes, primarily those belonging to the major histocompatibility complex (HLA), with more than 75 loci associated with its development, accounting for approximately 50% of all involved genetic systems [4]. According to the American Diabetes Association (ADA), autoimmune diabetes mellitus is associated with HLA haplotypes of the DQB1 and DRB1. The most well–known predisposing haplotypes are DRB10301-DQB10201 (DR3–DQ2) and DRB10401-DQB10302 (DR4-DQ8). There are genetic differences in the risk of T1DM across various populations. In the Russian Federation, the strongest predisposing HLA-DR and DQ haplotypes are considered to be DRB1*04-DQA1*03,01-DQB1*03,02 and DRB1*04-DQA1*03,01-DQB1*03,04; intermediate-risk haplotypes include DRB1*17(03)-DQA1*05,01-DQB1*02,01, DRB1*01-DQA1*01,01-DQB1*05,01, and DRB1*16-DQA1*01,02-DQB1*05,02/4; protective haplotypes include DRB1*15-DQA1*01,02-DQB1*06,02/8 and DRB1*13-DQA1*01,03-DQB1*06,02/8 [4,5].

Against the backdrop of rising T1DM incidence, comparative analyses of various factors reveal a declining relative contribution of the strongest HLA haplotypes and an increasing role of epigenetic factors, underscoring the need for their detailed investigation.

Epigenetic factors, their involvement in various signaling pathways, and the associated effects represent promising avenues of research in medicine. Epigenetic regulation of gene expression involving microRNAs-one of the most common epigenetic mechanisms-plays a crucial role in governing processes such as cell differentiation, proliferation, apoptosis, immunity, and homeostasis. MicroRNAs are small, single-stranded RNA molecules consisting of 19–25 nucleotides that do not encode proteins but modulate protein levels through post-transcriptional mechanisms by binding to the 3′-untranslated region (3′-UTR) of target mRNAs, leading to mRNA degradation or translational repression [6,7,8]. The molecular mechanism of epigenetic regulation by microRNAs involves inhibition of translation by binding to the 3′-untranslated region (3′-UTR) of target mRNAs, leading to their cleavage by Drosha and Dicer enzymes from the RNase III family. Altered microRNA expression profiles are indicative of pathological conditions including diabetic nephropathy, retinopathy, and cardiovascular and cerebrovascular diseases [7,8,9]. Consequently, in recent years, microRNAs have been increasingly considered potential biomarkers for early diagnosis, progression assessment, and monitoring of disease complications. The presence of microRNAs in biological fluids (serum, blood plasma, saliva, urine) makes them readily accessible and minimally invasive diagnostic tools. Unlike RNA molecules that rapidly degrade under the action of ribonucleases, circulating microRNAs are stable due to protection by exosomes, lipoproteins, and RNA-binding proteins [1,10]. Furthermore, they are resistant to multiple freeze–thaw cycles, pH fluctuations, and high temperatures [10]. Highly sensitive and specific techniques, such as microarray technology, quantitative reverse transcription polymerase chain reaction (qRT-PCR), rolling circle amplification (RCA), ligase chain reaction (LCR), and biosensor technologies, enable rapid, accurate, and efficient real-time quantification of specific microRNA levels in clinical specimens [9].

Collectively, the above considerations suggest that in the near future, microRNAs will be used not only to establish specific diagnostic signatures for various diseases but also as effective therapeutic agents. Modulating microRNA behavior or correcting aberrant expression levels either by introducing exogenous microRNA molecules or by inhibiting their function represents a promising therapeutic strategy [11].

In this context, diseases associated with dysregulated metabolism represent some of the most promising research directions, given that microRNAs govern central metabolic pathways, thereby maintaining energy balance and metabolic homeostasis. Traditional approaches to predicting the clinical stage of T1DM based on the quantification of diabetogenic autoantibodies do not reliably predict the progression and timing of the clinical onset of T1DM. The time before the onset of the disease can range from several months to several years after the appearance of autoantibodies to islet cells. The rate of progression of T1DM is likely to vary depending on age, family history, environmental factors, genetics, antigens affected by the immune system, and factors inherent in beta cells (for example, endoplasmic reticulum stress, improper folding of proinsulin, overexpression of HLA class I molecules on the cell surface). Thus, MicroRNAs are promising biomarkers that enable early diagnosis of the disease before clinical manifestations. This approach may allow for the timely initiation of therapy, particularly immunosuppression, before beta- cell degradation. Another advantage of using microRNA diagnostics is complication monitoring.

The objective of this review is to summarize current evidence on the role of microRNAs in the pathogenesis of T1DM and T2DM and to assess their potential as biomarkers and therapeutic targets.

The review was carried out using the PubMed, and SCOPUS databases. The following keywords and medical subject (MeSH) terms were used: “microRNA” AND (“diabetes melitus” OR “T1DM” OR “T2DM” OR “biomarkers of diabetes melitus” OR “glucose intolerance” OR “glycaemic index” OR “hyperglycaemia”). Inclusion criteria were defined according to the PICO framework: population—patients with diabetes mellitus (any type) or relevant animal models; intervention—assessment of microRNA expression levels; comparator—healthy controls, normoglycemic animals, or baseline expression; outcomes—diagnostic, prognostic, or mechanistic data on microRNAs in diabetes; study design—original research articles, including randomized controlled trials (RCTs), observational studies, clinical studies, clinical trials, and case reports. Only English-language articles with full-text availability were included. The following restrictions were applied in the review of the available literature: studies from the last 10 years, full free access and relevance to the search terms. Searches were limited to English-language full-text articles with the statistical analysis of experimental data carried out with a confidence level of *p* < 0.05 and below. Records were independently screened by at least two reviewers at successive stages of selection. Discrepancies were resolved through discussion and consensus, or by involving a third reviewer. After screening and full-text assessment, studies meeting the inclusion criteria and deemed methodologically sound were retained for qualitative synthesis and discussion.

## 2. The Role of MicroRNAs in the Pathogenesis of Diabetes Mellitus

Diabetes mellitus is a group of chronic metabolic disorders characterized by hyperglycemia resulting from defects in insulin secretion and/or the interaction of the hormone insulin with target cells (insulin resistance). There are several types of diabetes (T1D, T2D, GDM, monogenic diabetes, and several other rare specific forms of diabetes) [9,12,13,14]. The most prevalent are T1D and T2D, which, together with GDM, account for virtually 100% of disease cases; therefore, for these three types of diabetes, we will analyze the significance of aberrant microRNA expression. In advanced stages of T1DM the titer of autoantibodies may decrease or even be absent.

**Figure 1:**
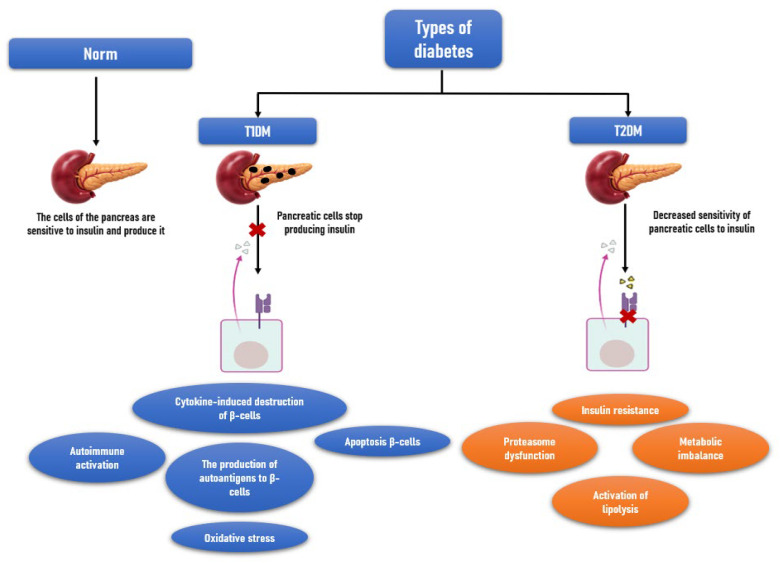
Mechanisms of disease development and their consequences.

T1DM is a multifactorial disease. Genetic predisposition, environmental factors (triggers of the pathological process), and the state of the immune system play a significant role in the development of T1DM [10]. The pathogenesis of T1DM involves a complex, multi-stage pathological process leading to autoimmune destruction of pancreatic β-cells, the main causes of which include activation of polyclonal lymphocytes, molecular mimicry, and increased immunogenicity. Following initial β-cell damage induced by triggers (infectious, nutritional, chemical, psychosocial), antigen presentation occurs, and the islets become infiltrated by autoreactive T-lymphocytes, leading to autoimmune activation and further β-cell injury. Subsequently, infiltration proceeds, and monocytes and macrophages secrete cytokines (interleukin 1(IL-1), interleukin 6 (IL-6), tumor necrosis factor-α (TNF-α)) as well as free oxygen radicals, nitric oxide, and hydroxyl radicals. Free radicals damage β-cell DNA, while cytokines induce apoptosis. Destruction occurs at varying rates and becomes clinically significant when approximately 70–90% of β-cells have been destroyed [4,15] (Figure 1).

Currently, T1DM is classified into four stages, Stage 1 is characterized by the presence of single autoantibodies against pancreatic islet cells, normal blood glucose levels, and the absence of clinical symptoms; Stage 2 is marked by multiple islet cell autoantibodies and β-cell dysfunction due to a progressive decline in β-cell mass, as well as impaired glucose tolerance; Stage 3 is defined by blood glucose levels rising above diagnostic thresholds and the onset of clinical symptoms; Stage 4 represents the established chronic form of T1DM [4,5]. Serological markers of autoimmune β-cell destruction include specific autoantibodies (Abs) against glutamate decarboxylase (GADA), tyrosine phosphatase (IA-2A), insulin (IAA), and zinc transporter 8 (ZnT8A) [5] It should be noted that in some cases, islet cell autoantibodies may be absent during the initial stage of the disease, and T1D may not develop in individuals who test positive for autoantibodies [16].

A hallmark of T2DM is insulin resistance—a systemic failure of insulin-mediated glucose uptake by target tissues, primarily the liver, skeletal muscle, adipose tissue, and brain cells [6]. Under normal physiological conditions, insulin binds to its receptor on the surface of these cells, triggering a signaling cascade that facilitates glucose transport into the cell via glucose transporter type 4 (GLUT4). In the state of insulin resistance, this signaling pathway is impaired, resulting in reduced glucose uptake and utilization by peripheral tissues. Consequently, blood glucose levels rise, creating a state of chronic hyperglycemia [3,5].

In response to this hyperglycemia, pancreatic β-cells initially compensate by increasing insulin production and secretion, leading to hyperinsulinemia [9]. Over time, however, the sustained demand placed on β-cells leads to their progressive dysfunction and eventual failure. This transition from compensatory hyperinsulinemia to inadequate insulin secretion marks the clinical progression from prediabetes to over T2DM [15].

### 2.1. Aberrant MicroRNA Expression Profile in Type 1 Diabetes Mellitus

It is known that T1DM often remains clinically asymptomatic for several months or even years. Therefore, novel biomarkers that assess not only the presence of pancreatic islet cell autoantibodies but also β-cell dysfunction are required to predict the onset of T1DM. In this regard, the analysis of epigenetic dysregulation represents a promising direction. To date, a considerable number of microRNAs exhibiting aberrant expression in T1DM have been identified [1]. The most frequently detected microRNAs with altered expression profiles, originating from both endocrine and immunological sources, may subsequently be utilized for early diagnosis, monitoring disease progression, and in clinical trials aimed at developing effective treatment protocols for T1DM.

Experimental studies are being conducted using animal models (primarily rodent models), biological fluids (serum, plasma), or tissue samples from patients with T1DM, cell cultures, peripheral blood mononuclear cells, and plasma-derived exosomes [1,9,10,17,18,19,20]. Unfortunately, the involvement of several microRNAs in T1DM pathogenesis has been confirmed in only one, or at most three, published studies, which is clearly insufficient to consider such microRNAs as significant disease signatures.

Differences in the expression of specific microRNAs may correspond to different stages of T1DM and correlate with disease severity [8,10,20,21], with aberrant microRNA levels potentially being either upregulated or downregulated relative to normal values. Additional challenges in interpreting the obtained results arise from emerging data indicating divergent expression patterns of certain T1DM-associated microRNAs across different tissues and/or species (e.g., human vs. mouse) [17,18]. Nevertheless, interest in this topic continues to grow, and research aimed at elucidating the mechanisms by which T1DM-specific microRNAs contribute to the onset and progression of the pathology is currently becoming a priority.

#### 2.1.1. miR-21

Circulating miR-21 appears to be a promising biomarker for T1DM. Molecules of this microRNA have been detected in plasma, serum, and peripheral blood mononuclear cells, consistently demonstrating elevated levels across several studies [1,10,19,22,23]. As shown in animal models, miR-21 impedes β-cell development [19] and, by regulating PI3K/AKT signaling and participating in *Bcl-2* gene translation, enhances β-cell apoptosis, leading to impaired insulin release during diabetes progression [10,24]. Furthermore, this microRNA is mentioned in several studies in the context of active autoimmune inflammation and/or immune cell function [8]. Importantly, elevated miR-21 levels are associated with increased HbA1c levels, indicating direct or indirect involvement in glycemic control mechanisms [25,26].

#### 2.1.2. miR-24-3p

Several studies have documented abnormally elevated levels of miR-24-3p in serum and plasma samples from individuals with T1DM, particularly in patients with substantial (>90%) C-peptide loss [8,27,28].

Furthermore, in experiments using mouse models, miR-24-3p demonstrated a positive correlation with osteoprotegerin (OPG), a protein that reduces insulitis, improves glucose homeostasis, and increases functional β-cell mass [29]. Conversely, miR-24-3p levels correlated negatively with soluble CD40 ligand (sCD40L), resistin, myeloperoxidase (MPO), and soluble tumor necrosis factor receptor (sTNFR)-markers associated with inflammation and β-cell dysfunction [8].

#### 2.1.3. miR-25

miR-25 is also considered a participant in key pathways involved in T1DM pathogenesis, being associated with inflammation, autoimmune processes, and β-cell defects. Its expression is significantly upregulated in plasma-derived exosomes and serum from individuals with T1DM [1,8,22,23,27]. miR-25 levels correlated negatively with the percentage of glycated hemoglobin (HbA1c) and positively with meal-stimulated C-peptide levels, which appears to reflect the influence of this microRNA on glucose metabolism and diabetes progression. Since aberrant miR-25-3p levels contribute to the death of insulin-producing β-cells via signaling cascades involving IL-1β and IRS-1, it is hypothesized that monitoring the expression profile of miR-25, which indicates the extent of β-cell damage, may aid in the development of therapeutic strategies aimed at preserving β-cell function [1,8].

#### 2.1.4. miR-92a and miR-126

Expression levels of miR-92a and miR-126 were significantly reduced in serum samples from patients with T1DM [10]. miR-92a appears to be involved in the regulation of NF-κB and other inflammatory pathways, thereby contributing to the development of diabetic complications, particularly cardiovascular disease [30]. miR-126 targets IL-1β and IRS-1, promoting β-cell death, and is critical for maintaining endothelial homeostasis, which is frequently disrupted in diabetes [10,19]. Furthermore, reduced expression of miR-126 has been associated with endothelial inflammation in individuals with microvascular and macrovascular complications of T1DM [10].

#### 2.1.5. miR-146a

miR-146a, which can target genes critical for the function and survival of insulin-producing β-cells, has demonstrated varying dysregulation patterns across different sample types in T1DM. Elevated expression of plasma miR-146a has been documented in several studies [8,31,32]. Moreover, circulating miR-146a levels exhibited an inverse correlation with C-peptide levels and autoimmunity [8]. However, opposite dynamics were observed in peripheral blood mononuclear cells, where miR-146a/b levels were markedly decreased in T1DM [1].

#### 2.1.6. miR-148a-3p

Elevated levels of miR-148a-3p were observed in plasma, serum, T cells, and peripheral blood mononuclear cells from patients with T1DM [23]. This microRNA, which is involved in the PI3K/AKT signaling cascade, regulates inflammation and β-cell apoptosis. Upregulation of miR-148a-3p, together with the involvement of PTEN and SOX6, leads to impaired insulin secretion [1].

#### 2.1.7. miR-375

miR-375 is mentioned as a promising circulating biomarker of β-cell death in T1DM [23]. This microRNA is considered a well-established regulator of β-cell mass and function. It inhibits genes responsible for insulin production and participates in glucose regulation by modulating insulin secretion [8,32]. Elevated levels of miR-375 have been detected in plasma, serum, and peripheral blood mononuclear cells [10,23]. However, decreased expression of circulating miR-375 has also been reported [33]. Given that the expression profile of miR-375-3p predicts fasting C-peptide levels over a 9-10-month period, miR-375 may be useful for monitoring the outcomes of certain immunotherapies in T1DM [8].

#### 2.1.8. miR-409-3p

Lower levels of miR-409-3p were observed in plasma samples from individuals with newly diagnosed T1DM [10]. miR-409-3p targets key T1DM autoantigens GAD65 and IA-2β, suggesting its important role in regulating autoimmune processes associated with T1DM [8]. Indeed, aberrant miR-409-3p levels indicate a poorer clinical prognosis for the disease [21] and demonstrate an inverse correlation with circulating HbA1c [20].

In summary of this section, it can be stated that analyzing the role of microRNAs most promising in terms of their involvement in T1DM pathogenesis, as well as identifying novel such biomarkers, will significantly enhance our understanding of the fundamental molecular mechanisms underlying this disease and will facilitate more personalized approaches to the management of patients with T1DM, potentially blocking or slowing its progression. Given that current treatment modalities for T1DM only alleviate symptoms but do not prevent β-cell destruction, leaving patients dependent on lifelong insulin therapy, such research is of particular importance.

Among T1DM-associated microRNAs (Table 1), the most frequently reported proposed functions include β-cell dysfunction/apoptosis, inflammation/autoimmunity, insulin secretion, endothelial homeostasis, and glucose homeostasis. Notably, several miRNAs (miR-21, miR-25, miR-146a, miR-148a-3p) are implicated in both β-cell dysfunction and autoimmune pathways, suggesting their potential dual role in T1DM pathogenesis. All T1DM-related data were derived from patient samples, with PBMCs, plasma, and serum representing the most common biological sources.

### 2.2. Aberrant MicroRNA Expression Profile in Type 2 Diabetes Mellitus and Obesity

T2DM is a complex metabolic disorder characterized by impaired insulin signaling and glucose homeostasis, accompanied by abnormal lipid metabolism [34]. Excess body weight correlates with disease progression and an increased risk of complications. A hallmark of T2DM is insulin resistance—a systemic failure of insulin-mediated glucose uptake by target tissues, primarily the liver, skeletal muscle, adipose tissue, and brain cells, which in turn stimulates increased insulin production by β-cells and leads to hyperinsulinemia. The resulting chronic hyperglycemia progresses to T2DM. T2DM primarily affects adults, but due to rising obesity rates and sedentary lifestyles, it is increasingly being diagnosed in younger populations as well [35]. T2DM accounts for approximately 90–95% of all diabetes cases [4], with a higher prevalence in men than in women [36]. Age represents an additional risk factor for T2DM development, after 75 years, the incidence increases by nearly one-quarter [9]. Unhealthy diet, smoking, alcohol consumption, and poor environmental conditions also contribute to the onset and progression of this disease. Furthermore, genetic factors contribute to the etiology of T2DM [37]. Numerous lines of evidence have emerged supporting the involvement of epigenetic mechanisms in the evolution of T2DM [38]. Finally, liver dysfunction, thyroid disorders, Cushing’s syndrome, hypertension, and metabolic syndrome play a role in the pathogenesis of T2DM and the development of associated complications [39,40]. T2DM is the most rapidly growing metabolic disease worldwide; over the past three decades, the number of T2DM cases globally has doubled [40].

T2DM develops slowly over several years and is initially asymptomatic. Traditional diagnostic approaches only detect hyperglycemia—a condition that manifests at a later stage of T2DM pathogenesis—and fail to capture the early stages of the disease. However, evidence has emerged indicating that the expression profiles of certain microRNAs are altered in biological fluids several years prior to the clinical manifestation of T2DM [41,42]. It is evident that microRNAs involved in the regulation of cellular functions, insulin secretion, and insulin signaling pathways offer a broad range of opportunities not only for early detection of T2DM but also for effective monitoring of disease progression. Identifying specific microRNAs with characteristic expression abnormalities and understanding the mechanisms by which such microRNAs participate in T2DM pathogenesis will subsequently help establish a list of promising therapeutic targets for combating this pathology.

To date, a considerable number of microRNAs associated with the pathophysiology of T2DM have been identified. These microRNAs control pancreatic β-cell function and insulin secretion, participate in adipocyte differentiation and function, and contribute to the development of insulin resistance in various target tissues, including the liver, skeletal muscle, and adipose tissue.

Below, we discuss the most significant and well-studied microRNAs in the context of T2DM onset and progression.

#### 2.2.1. miR-1

miR-1 demonstrates decreased expression in skeletal muscle under conditions of insulin resistance, and dysregulation of this microRNA’s expression profile has been documented in both “in vitro” and “in vivo” studies [38].

#### 2.2.2. miR-9

miR-9, expressed in pancreatic β-cells, reduces glucose-stimulated insulin secretion by targeting SIRT1 (sirtuin 1) expression, indicating its involvement in the pathogenesis of T2DM [6]. Studies in animal models have shown that miR-9 can regulate insulin release through interaction with the transcription factor OC2 (onecut-2), a negative regulator of granuphilin SLP4/SYTL4, which is associated with β-cell secretory granules. Basal expression of miR-9 is required to maintain appropriate granuphilin levels and optimal insulin secretory capacity in β-cells. Elevated miR-9 levels lead to reduced glucose-stimulated insulin secretion in β-cells [43]. Furthermore, among microRNAs involved in the regulation of lipid metabolism and insulin sensitivity, miR-9 has attracted researchers’ attention due to its role in hepatic glucose homeostasis [39]. miR-9 participates in the regulation of enzymes such as PEPCK and G6Pase, as well as the transcription factor FOXO1, which are critical for gluconeogenesis. Downregulation of miR-9 is accompanied by increased FOXO1 expression in the liver and activation of hepatic gluconeogenesis, leading to obesity [38]. Thus, the functional activity of miR-9 may vary significantly during different stages of T2DM development, making this microRNA an interesting subject for further research and a potential biomarker candidate. Periodic profiling of miR-9 levels is likely to provide valuable insights into this pathology.

#### 2.2.3. miR-10b-5p

The expression level of miR-10b-5p decreases during adipogenic differentiation [44] and remains low in adipose tissue, indicating the involvement of this microRNA in the regulation of adipocyte function [38]. Furthermore, miR-10b-5p demonstrates reduced expression in skeletal muscle under conditions of insulin resistance [38].

#### 2.2.4. miR-15

In the context of analyzing T2DM onset, the miR-15 family is of considerable interest. Members of this family are involved in insulin biosynthesis and secretion and appear to play a role in the regulation of adipocyte function. In β-cells, miR-15a inhibits the UCP2 protein, acting as a negative regulator of insulin secretion [6]. The level of this microRNA is reduced in the serum of individuals with obesity and T2DM [6,44]. However, expression of miR-15, which promotes increased insulin production [6], is upregulated during adipogenic differentiation [44]. These microRNAs are also being considered as potential biomarkers for assessing the risk of T2DM development and as promising therapeutic targets in obesity [6].

#### 2.2.5. miR-17

miR-17 demonstrates increased expression in skeletal muscle under conditions of insulin resistance [38]. Elevated miR-17 levels were observed in muscle tissue of rats with T2DM, whereas downregulation of miR-17 led to increased levels of the glucose transporter GLUT4 protein and was accompanied by improved glucose metabolism [45].

#### 2.2.6. miR-21

The expression level of miR-21, which is associated with the regulation of FOXO1 and PTEN, is reduced in insulin-resistant adipocytes and in the plasma of patients with T2DM [6,44]. Downregulation of this microRNA enhances insulin signaling in liver and adipose tissue cells and is involved in the pathogenesis of obesity [6]. Studies in mice fed a high-fat diet and in obese patients demonstrate that miR-21 expression correlates inversely with PTEN levels [46]. Similarly, upregulation of miR-21 suppresses hepatic glycogenesis and reduces the expression of the transcription factor FOXO1, which is typically elevated in the liver of obese individuals [38]. Analyzing the dysregulation of miR-21 expression in patients with T2DM may provide valuable insights into the body’s response to elevated blood glucose levels [12].

#### 2.2.7. miR-23a-3p

miR-23a-3p plays an important role in regulating glucose homeostasis and insulin sensitivity in adipose tissue [47]. Dysregulation of this microRNA can significantly alter adipocyte function, contributing to obesity and insulin resistance [38]. Indeed, miR-23a-3p expression is markedly reduced in the adipose tissue of obese mice [47].

#### 2.2.8. miR-24

miR-24 levels are reduced in the plasma of patients with T2DM [44]. Additionally, miR-24-3p demonstrates decreased expression in skeletal muscle under conditions of insulin resistance [38].

#### 2.2.9. miR-26a

In individuals with T2DM and insulin-resistant mice, decreased levels of miR-26a are observed in the liver [48]. miR-26a is involved in the regulation of insulin sensitivity by controlling the expression of key metabolic genes, including Gsk3b, Pepck (Pck1), and members of the protein kinase C enzyme family [48]. In experimental models using mice fed a high-fat diet, upregulation of miR-26a improved insulin sensitivity and was accompanied by reduced hepatic glucose production and lipogenesis. miR-26a also influences adipocyte differentiation, as its expression increases during adipogenic differentiation [44].

#### 2.2.10. miR-27

miR-27, by regulating the expression of critical components of the insulin signaling pathway, namely INSR (insulin receptor) and IRS (insulin receptor substrates), influences insulin resistance and is involved in the pathogenesis of T2DM [6]. Members of the miR-27 family act as negative regulators of adipocyte differentiation [6,49]. In particular, overexpression of miR-27b suppresses the expression of PPARγ, a key regulator of lipid homeostasis during adipogenic differentiation, as well as the transcription factor C/EBPα, thereby inhibiting adipogenesis in the liver [49]. Furthermore, miR-27a affects insulin signaling and demonstrates increased expression in skeletal muscle under conditions of insulin resistance [38]. Analyzing the dysregulation of miR-27 family members in patients with T2DM may provide important insights into the body’s response to elevated blood glucose levels [12].

#### 2.2.11. miR-28-3p

miR-28-3p levels are elevated in the serum of patients with T2DM, and alterations in the expression profile of this microRNA were observed prior to the clinical manifestation of the disease [44].

#### 2.2.12. miR-29

Expression levels of members of the miR-29 family are critical markers of β-cell function and regulators of insulin secretion and lipid metabolism [8,36]. Expression of miR-29a, miR-29b-1, miR-29b-2, and miR-29c-3p is dysregulated in metabolic diseases, obesity, and insulin resistance [40]. Members of the miR-29 family are involved in insulin signaling and glucose homeostasis in the liver, influencing the AMPK and PGC-1α signaling pathways, which play central roles in energy metabolism and mitochondrial biogenesis [34]. miR-29b levels are reduced in individuals with prediabetes and T2DM [1]. Forced expression of miR-29 in the liver of diabetic mice significantly reduced hyperglycemia [48]. miR-29 expression is elevated in adipose tissue in T2DM [38]. It has been found that members of this microRNA family downregulate the expression of SPARC (secreted protein acidic and rich in cysteine) and GLUT4, negatively affecting glucose uptake in adipocytes [38]. miR-29a demonstrates increased expression in skeletal muscle under conditions of insulin resistance [38]. Additionally, miR-29 is indirectly involved in inflammatory processes in pancreatic islet β-cells through TRAF3 (TNF receptor-associated factor 3) signaling, enhancing the release of the proinflammatory chemokine Cxcl10 (C-X-C motif chemokine ligand 10) from β-cells and promoting the recruitment of monocytes and macrophages [38]. Thus, accumulating evidence indicates a significant role of the miR-29 family in the pathogenesis of prediabetes and T2DM [50]. Consequently, members of this family are considered among the most promising potential biomarkers and therapeutic targets for suppressing inflammation and treating T2DM [38].

#### 2.2.13. miR-30

The miR-30 family is another group of microRNAs important for the onset and progression of T2DM. Changes in the expression levels of members of this family may be bidirectional depending on the microRNA source. Specifically, expression levels of miR-30a-5p are reduced in adipose tissue from individuals with T2DM compared to healthy controls [51]. However, higher circulating levels of miR-30a-5p have been documented in T2DM [52]. Initially, it was established that miR-30c and miR-30a-5p, which targets BDNF (brain-derived neurotrophic factor), a key regulator of appetite [12], are involved in adipogenesis and obesity [44]. Currently, it is known that in addition to miR-30a-5p, the expression of three other members of the miR-30 family is also reduced in the adipose tissue of individuals with T2DM [51]. Suppression of these microRNAs in adipocytes leads to altered expression of genes involved in carbohydrate and lipid metabolism that are associated with insulin resistance and T2DM, in particular, to reduced phosphorylation of TBC1D4, a protein that regulates glucose transport in skeletal muscle and adipose tissue, and consequently, to decreased glucose uptake [51]. It has been established that miR-30d directly and/or indirectly regulates GLUT4 expression [47]. Experiments using the cultured pancreatic β-cell line MIN6 demonstrated that under conditions of sustained high glucose concentration, miR-30d expression levels increased, correlating with increased insulin gene expression, but this was not accompanied by enhanced insulin secretion. It appears that targets of miR-30d include negative regulators of insulin production [44]. Members of the miR-30 family are also considered potential biomarker candidates for T2DM, capable of contributing to the prevention of disease in individuals at high risk [52,53].

#### 2.2.14. miR-33

miR-33 plays a notable role in hepatic lipid homeostasis and glucose metabolism. This microRNA, which targets the INSR and IRS2 genes, alters insulin resistance by reducing insulin signaling in hepatocytes and is involved in the pathogenesis of T2DM [6]. By targeting SREBP-1c and FAS, key enzymes in lipogenesis, miR-33 participates in cholesterol metabolism and fatty acid oxidation [34]. Furthermore, by targeting HMGA2 (high mobility group AT-hook 2), miR-33 inhibits adipogenesis [54].

#### 2.2.15. miR-34a

miR-34a expression is significantly elevated in the serum and subcutaneous adipose tissue of individuals with obesity and T2DM [48]. Dysregulation of miR-34-5p has also been observed in children with obesity and insulin resistance [55]. This microRNA appears to be involved in the regulation of adipocyte function and modulates inflammatory processes by directly or indirectly controlling the release of proinflammatory cytokines from adipocytes and macrophages [44,48]. miR-34c levels increase during adipogenic differentiation [44], whereas complete knockout of miR-34a leads to increased susceptibility to the effects of a high-fat diet and rapid obesity onset [48].

#### 2.2.16. miR-103 and miR-107

miR-103 and miR-107 are also involved in adipogenesis, obesity, and insulin resistance, making a notable contribution to the pathophysiology of T2DM [44]. The expression levels of these microRNAs are elevated in T2DM [12].

miR-103 and miR-107 act as negative regulators of insulin signaling in hepatocytes, and their expression is increased in the liver of obese mice [6,56]. It is hypothesized that increased secretion of exosomal miR-103-3p by adipocytes may act as a driver of hepatic hyperinsulinemia [56].

miR-103 and miR-107 suppress the differentiation of white adipocytes, which is part of the mechanism by which these microRNAs contribute to the development of insulin resistance [48]. miR-103 and miR-107 influence insulin sensitivity by regulating Cav-1 (caveolin-1), an important component of the INSR signaling cascade. In adipocytes, downregulation of these microRNAs activates Cav-1 expression, stabilizes INSR, leading to enhanced insulin signaling and increased insulin-stimulated glucose uptake [6,44]. miR-103 and miR-107 have been identified as potential circulating biomarkers for T2DM [12].

#### 2.2.17. miR-122

The level of miR-122, which is abundantly expressed in hepatocytes, is elevated in patients with T2DM compared to healthy individuals. These microRNAs are involved in the pathophysiology of T2DM by aberrantly regulating hepatic lipid homeostasis, glucose metabolism, and inflammation-related signaling cascades [34,57]. Elevated miR-122 levels inhibit the expression of PTP1B (protein tyrosine phosphatase 1B), which dephosphorylates INSR and IRS, thereby enhancing insulin signaling in hepatocytes [6]. Expression of liver-specific miR-122 is markedly reduced upon overexpression of the protein kinase JNK1, which plays an important role in the development of obesity and insulin resistance. Accordingly, inhibiting JNK1 can increase miR-122 expression and, consequently, reduce insulin resistance [58]. Numerous hepatic nuclear receptors positively regulate miR-122 transcription. The best-characterized regulators include HNF4α (hepatocyte nuclear factor 4α), RAR-related orphan receptor alpha, and farnesoid X receptor [48]. Circulating miR-122 expression is elevated in patients with fatty liver disease and in obese individuals [48]. Suppression of miR-122 expression in vivo improved lipid and cholesterol profiles in wild-type and obese mice [48]. It appears that miR-122 reduces the rate of lipid accumulation by modulating genes involved in fatty acid synthesis and oxidation [34]. Finally, it should be noted that dysregulation of miR-122-5p has also been observed in children with obesity and insulin resistance [55]. Collectively, all the above suggests the potential application of this microRNA as a biomarker for T2DM and associated comorbidities.

#### 2.2.18. miR-124

miR-124 is involved in glucose metabolism, insulin secretion, and β-cell differentiation [6]. Increased expression of miR-124a leads to excessive insulin release under basal conditions and reduces glucose-stimulated insulin secretion [38]. Experiments using the cultured pancreatic β-cell line MIN6 demonstrated that elevated miR-124a expression levels are observed under high glucose conditions [44]. Additionally, miR-124a2 expression is significantly increased during late stages of β-cell differentiation. This isoform of miR-124a, which targets the Fox family transcription factor FOXA2, essential for glucose homeostasis, is involved in the insulin signaling pathway and acts as a negative regulator of insulin secretion [6]. It is hypothesized that miR-124a, along with other microRNAs involved in the early stages of T2DM development, may eventually become biomarkers of prediabetes [59].

#### 2.2.19. miR-125b-5p

In mouse models, it has been found that in T2DM, miR-125b-5p expression is decreased, accompanied by increased expression of JNK kinase, which is involved in inflammatory signaling. Conversely, increased expression of miR-125b-5p, by inhibiting the JNK signaling pathway, enhances insulin sensitivity and improves pancreatic β-cell function [38].

#### 2.2.20. miR-126-3p

miR-126 expression levels are lower in individuals with prediabetes and T2DM compared to healthy controls [6]. It is hypothesized that this microRNA is involved in T2DM pathogenesis through inflammatory pathways [57]. miR-126 influences insulin resistance by regulating INSR and IRS [1]. Along with other microRNAs targeting the IRS1 gene, miR-126 reduces insulin signaling in hepatocytes [6]. As a negative regulator of IRS-1, miR-126 impairs Lunapark function. The resulting endoplasmic reticulum stress due to decreased Lunapark levels leads to exacerbated insulin resistance and alters glucose homeostasis, thereby contributing to the pathophysiology of T2DM [60]. Analysis of the mechanisms underlying miR-126 expression dysregulation in T2DM may be useful for predicting diabetes development [6,12].

#### 2.2.21. miR-132

miR-132 plays a certain role in metabolic disorders associated with abnormal responses to insulin signaling. Circulating miR-132 expression is reduced in obese individuals [6]. The effects of miR-132 on the metabolic regulators FOXO3, Ep300, SIRT1, and Pten induce fatty liver disease and insulin resistance in mice [61]. These abnormalities may be corrected through epigenetic modulation [62]. miR-132 is being considered as a potential circulating biomarker for T2DM [12].

#### 2.2.22. miR-133a

The isoforms miR-133a-3p and miR-133b-3p regulate the expression of the glucose transporter GLUT4, a key player in insulin-stimulated glucose uptake in muscle and adipose tissues [38]. Decreased GLUT4 expression is observed in prediabetes and T2DM [53]. miR-133 acts as a negative regulator of glucose uptake. Reduced miR-133a expression levels have been observed in skeletal muscle under conditions of insulin resistance [38]. Furthermore, this microRNA is known to be involved in the pathogenesis of cardiovascular complications [6]. At the current stage of knowledge regarding the regulatory mechanisms of miR-133a, there is insufficient evidence to predict the use of this microRNA as a biomarker for T2DM.

#### 2.2.23. miR-135a

miR-135a demonstrates increased expression in skeletal muscle under conditions of insulin resistance [38]. miR-135, which regulates the INSR, IRS2, and PI3K/AKT signaling pathways, reduces insulin signaling in skeletal muscle and is involved in the pathogenesis of T2DM [6].

#### 2.2.24. miR-142-3p

Circulating miR-142-3p levels are elevated in obesity, with this increase being particularly significant in children with obesity [55]. miR-142-3p is considered a promising biomarker for T2DM [12].

#### 2.2.25. miR-143

miR-143 is involved in adipogenesis and obesity [44]. Circulating miR-143 levels are elevated in obesity, including in children [55]. miR-143, which targets the adipocyte-specific genes *GLUT4*, *aP2*, *HSL*, and *PPAR-γ2*, regulates adipocyte differentiation and adipogenesis. Upregulation of miR-143 suppresses white adipocyte differentiation and appears to be part of the mechanism by which these microRNAs contribute to the development of insulin resistance [63]. miR-143 negatively affects insulin signaling in hepatocytes by disrupting PKD/AKT signaling [6]. Increased miR-143 expression levels have been documented in the liver of diabetic mice [48]. Knockout of miR-143 in mice improved insulin sensitivity by enhancing brown adipose tissue thermogenesis and suppressing white adipose tissue adipogenesis. This resulted in a significant reduction in body weight, increased energy expenditure, decreased insulin resistance, and improved glucose tolerance [64]. Thus, in adipose tissue, miR-143 represents a promising target for epigenetic therapy in the treatment of obesity [48].

#### 2.2.26. miR-146

Members of the miR-146 family are also involved in processes associated with the development of T2DM. Circulating miR-146a expression levels are decreased in patients with T2DM compared to healthy individuals. This microRNA may potentially disrupt inflammatory signaling in metabolic diseases [57]. Dysfunction of the transcription factor FOXO1, a critical player in adipogenesis and insulin resistance, is linked to diabetes and obesity. FOXO1 levels are elevated in the liver of obese individuals. Increased expression of miR-146b reduces FOXO1 expression, thereby suppressing hepatic glycogenesis and insulin signaling [65]. Further studies of the miR-146 family are required to elucidate the mechanisms by which they influence the pathophysiology of T2DM.

#### 2.2.27. miR-150

Higher levels of miR-150 are observed in individuals with T2DM compared to healthy controls. These microRNAs may be considered potential biomarkers for assessing the risk of developing this pathology [52].

#### 2.2.28. miR-155

Patients with T2DM exhibit decreased miR-155 expression in serum [38,40]. Circulating miR-155 plays a crucial role in maintaining β-cell function [38]. Disruption of this mechanism may represent a key event in the transition from prediabetes to T2DM. miR-155 negatively affects adipogenesis, by inhibiting C/EBPβ expression, this microRNA impairs the development of brown and beige adipocytes. Accordingly, decreased miR-155 expression promotes obesity [48]. miR-155 regulates insulin sensitivity in the liver, adipose tissue, and skeletal muscle [40]. In particular, miR-155 levels are reduced in skeletal muscle under conditions of insulin resistance [38]. It is hypothesized that decreased miR-155 expression in patients with T2DM may be one of the major factors underlying the development of insulin resistance [66].

#### 2.2.29. miR-181

Members of the miR-181 family play an important role in adipogenesis and the regulation of adipocyte function, and also influence glucose homeostasis and insulin sensitivity in adipose tissue [38]. Expression of miR-181b [67]. miR-181a levels decrease during adipogenic differentiation [44]. An inverse correlation has been identified between miR-181a expression and adiponectin concentration [44]. Additionally, expression of miR-181b and miR-181a-5p is reduced in the adipose tissue of obese mice [47,67]. All of the above suggests the potential involvement of the miR-181 family in the development of T2DM and associated metabolic disorders.

#### 2.2.30. miR-182

miR-182 appears to be involved in the regulation of adipocyte function [44]. miR-182 expression levels decrease during the early stages of adipocyte differentiation, as well as in the adipose tissue of obese individuals [56]. Conversely, overexpression of miR-182 exerts an anti-adipogenic effect [56]. Furthermore, miR-182 demonstrates decreased expression in skeletal muscle under conditions of insulin resistance [38].

#### 2.2.31. miR-183

The target of miR-183 is *IRS-1*, indicating the involvement of miR-183 in the regulation of adipogenesis and insulin signaling [56]. Indeed, miR-183 levels increase during adipogenic differentiation [44]. Moreover, by suppressing *IRS-1* expression, miR-183-5p inhibits insulin signaling and insulin-induced glycogen synthesis in hepatocytes [64]. It appears that through this mechanism, miR-183-5p is involved in the pathophysiology of obesity-related hepatic insulin resistance [68].

#### 2.2.32. miR-184

miR-184, which regulates β-cell proliferation and differentiation, is involved in the early stages of T2DM development [38]. miR-184-3p directly targets the transcription factor CREB and regulates the transcriptional coactivator CRTC1, protecting β-cells from lipotoxicity and inflammation-induced apoptosis. miR-184-3p expression is reduced in pancreatic islets of patients with T2DM [40]. Conversely, the level of this microRNA is elevated in adipose tissue, suggesting its involvement in the regulation of adipocyte function [38]. miR-184-3p may be considered a potential biomarker for T2DM and obesity [59].

#### 2.2.33. miR-191

The expression level of circulating miR-191 is reduced in the plasma of patients with T2DM [44].

#### 2.2.34. miR-194

miR-194, which targets AKT and GSK3β, is involved in the insulin signaling pathway and negatively affects glucose metabolism in skeletal muscle [6]. Indeed, miR-194 expression levels decrease in skeletal muscle under conditions of insulin resistance [38].

#### 2.2.35. miR-197

miR-197 expression levels are reduced in the plasma of patients with T2DM [44].

#### 2.2.36. miR-200

The miR-200 family, comprising miR-200a, miR-200b, and miR-200c, is expressed in pancreatic islet cells and regulates β-cell survival and insulin secretion [69]. Dysregulation of miR-200-mediated mechanisms may lead to the development of T2DM. Overexpression of miR-200c in the cultured EndoC-βH1 cell line leads to decreased glucose-stimulated insulin secretion [17]. Additionally, miR-200c suppresses insulin exocytosis and contributes to reduced insulin secretion in human pancreatic islet cells [38]. Accordingly, downregulation of miR-200c increases glucose-stimulated insulin secretion approximately threefold in pancreatic islets from patients with T2DM [17].

#### 2.2.37. miR-202, miR-214

In skeletal muscle under conditions of insulin resistance, expression levels of miR-202 and miR-214 are markedly increased [38].

#### 2.2.38. miR-206

Conversely, miR-206 demonstrates decreased expression in skeletal muscle under conditions of insulin resistance [38].

#### 2.2.39. miR-223

miR-223 expression exhibits bidirectional patterns across different tissues in T2DM and concomitant obesity. This may explain why this microRNA is considered a tissue-specific biomarker [12]. miR-223 levels are reduced in the serum of patients with T2DM, and alterations in the expression profile of this microRNA were observed prior to clinical manifestation of the disease [44]. Conversely, miR-223 expression is elevated in adipose tissue [38]. It is thought that miR-223-3p directly and/or indirectly regulates the expression of the glucose transporter GLUT4, which plays an important role in insulin-stimulated glucose uptake in muscle and adipose tissues [38]. Given that miR-223 levels are lower in individuals with prediabetes or T2DM, its significance for predicting diabetes development is under discussion [6].

#### 2.2.40. miR-320

Similar to miR-223, miR-320 expression is reduced in the serum of patients with T2DM but increased in adipose tissue [38]. This microRNA reduces insulin sensitivity in adipocytes and is involved in the pathogenesis of obesity [6]. In adipocytes, miR-320 has been identified as an important regulator of insulin resistance. miR-320 expression in insulin-resistant adipocytes exceeded basal levels by 50-fold. miR-320 participates in the regulation of insulin resistance by targeting p85 kinase, leading to increased AKT phosphorylation and enhanced GLUT4 expression, which in turn potentiates PI3K insulin signaling [40]. Inhibition of miR-320 improves insulin sensitivity [6]. In addition to adults, dysregulation of miR-320a has also been observed in children with obesity and insulin resistance [55]. Since alterations in the expression profile of miR-320a were observed prior to the clinical manifestation of T2DM, this microRNA represents a good candidate biomarker for early stages of disease development [44].

#### 2.2.41. miR-335

It is likely that miR-335 may be indirectly involved in the pathophysiology of T2DM. miR-335 is associated with inflammation and metabolic dysregulation in adipose tissue, and this process is regulated by TNF-α [70]. TNF-α increases miR-335 expression in adipocytes, leading to decreased expression of genes involved in insulin signaling and lipid metabolism. Dysregulated microRNA expression in obesity and metabolic syndrome also correlates with low adiponectin levels [8]. Elevated miR-335 levels in adipose tissue suggest the involvement of this microRNA in adipose tissue hyperplasia [71].

#### 2.2.42. miR-375

miR-375 is an evolutionarily conserved microRNA specific to pancreatic islets. miR-375 is involved in the regulation of insulin secretion, β-cell development, and glucose homeostasis [44]. Aberrant blood levels of miR-375 have been documented up to five years prior to the development of prediabetes and T2DM [52]. Lower circulating miR-375 levels from adipose tissue have been documented in individuals with T2DM and insulin resistance [38]. However, experiments in mouse models of diabetes have shown that miR-375 expression is, conversely, elevated in pancreatic islets. A similar pattern is observed in individuals with T2DM [50]. By regulating CAV-1 expression, miR-375 plays an important role in β-cell development [72].

Overexpression of miR-375 leads to a reduction in β-cell number, decreases their viability, and impairs their sensitivity to glucose-stimulated insulin secretion [44]. Notably, the decline in insulin secretion in human pancreatic islet cells is an important phenotype observed in patients with T2DM [38]. Acting at the late stage of insulin exocytosis, miR-375 controls insulin secretion by regulating the PI3K/protein kinase B signaling cascade and inhibiting PDK1 (phosphoinositide-dependent protein kinase 1) [6]. Reduced levels of PDK1, a direct target of miR-375, contribute to decreased insulin gene expression in response to glucose stimulation [38]. Consequently, insulin-induced phosphorylation of AKT and GSK3 is reduced [6]. Loss of PDK1 in β-cells promotes progressive hyperglycemia due to decreased pancreatic islet density [73]. Another target of miR-375 is the protein Mtpn (myotrophin), which facilitates the fusion of insulin vesicles with β-cell membranes [44]. Mtpn also acts in the nucleus as a transcription factor activating NF-κB, a critically important component for maintaining glucose-stimulated insulin secretion in β-cells [44]. By activating NF-κB, Mtpn induces the expression of proteins responsible for the migration of insulin vesicles to the membrane [38]. An additional mechanism involving miR-375 limits the activation of PDX1 (pancreatic and duodenal homeobox-1) by glucose via the PI3-kinase signaling pathway [38].

miR-375 is being considered as a potential circulating biomarker for T2DM [12]. It is also thought that these microRNAs will improve prediction and facilitate the identification of individuals at high risk of developing diabetes [52].

#### 2.2.43. miR-486

miR-486 expression levels are reduced in the plasma of patients with T2DM [44].

#### 2.2.44. miR-495-3p

miR-495-3p expression is reduced in adipose tissue [38].

#### 2.2.45. miR-802

Elevated levels of miR-802 in the liver of obese mice enhance insulin activation of the PKB/AKT signaling pathway and, as a consequence, negatively affect insulin sensitivity and contribute to hepatic steatosis [6]. miR-802 is directly regulated by two nuclear receptors, FXR (farnesoid X receptor), which is involved in fat and carbohydrate metabolism, and SHP (small heterodimer partner), which functions as a corepressor [74]. SHP suppresses the expression of the key gluconeogenic genes *PEPCK* and *G6Pase*, thereby inhibiting hepatic glucose production. Additionally, miR-802 targets HNF1b (hepatocyte nuclear factor-1 beta), the loss of which leads to diabetes in young individuals [48].

#### 2.2.46. Let-7

Members of the let-7 family are involved in the regulation of glucose metabolism in the pancreas, liver, and skeletal muscle, as well as in adipocyte function [48]. Altered levels of circulating let-7 microRNAs have been observed in obesity and metabolic syndrome [56]. let-7a-3 expression was reduced in the adipose tissue of subjects with T2DM compared to the control group [51]. It appears that let-7 targets key genes of the insulin signaling pathway, including *INSR*, *IGF1R*, and *IRS2* [75]. By suppressing the expression of IR (insulin receptor), IGF-1 receptor, and IRS2, let-7 negatively regulates the AKT-mediated insulin signaling cascade in hepatocytes [48,56]. Furthermore, members of the let-7 family influence insulin secretion and insulin resistance through interaction with the RNA-binding protein Lin28 [48]. Experiments in mouse models have shown that overexpression of let-7 in the pancreas or skeletal muscle promotes the development of insulin resistance and impairs glucose tolerance. Conversely, mice with muscle-specific overexpression of Lin28 fed a high-calorie diet exhibited improved glucose metabolism and depletion of let-7 [76].

let-7b levels increase during adipogenic differentiation [44]. It is hypothesized that increased secretion of exosomal let-7 by adipocytes may act as a driver of hepatic insulin resistance under hyperinsulinemic and prediabetic conditions [56].

Since epigenetic disturbances in glucose homeostasis involving let-7 are reversible, members of this family are considered promising targets for use in preclinical models aimed at modulating carbohydrate metabolism programs.

As shown in the table (Table 2), the majority of microRNAs linked to T2DM have been studied in patient-derived biological samples, including plasma, serum, adipose tissue, skeletal muscle, and liver. Experimental models include both human subjects and animal models, with a strong emphasis on tissues central to glucose metabolism and insulin sensitivity. Notably, several T2DM-associated microRNAs (e.g., miR-29, miR-34a, miR-103/107, miR-122, let-7) are implicated in pathways related to insulin resistance, inflammation, and lipid metabolism.

## 3. Discussion

**Figure 2:**
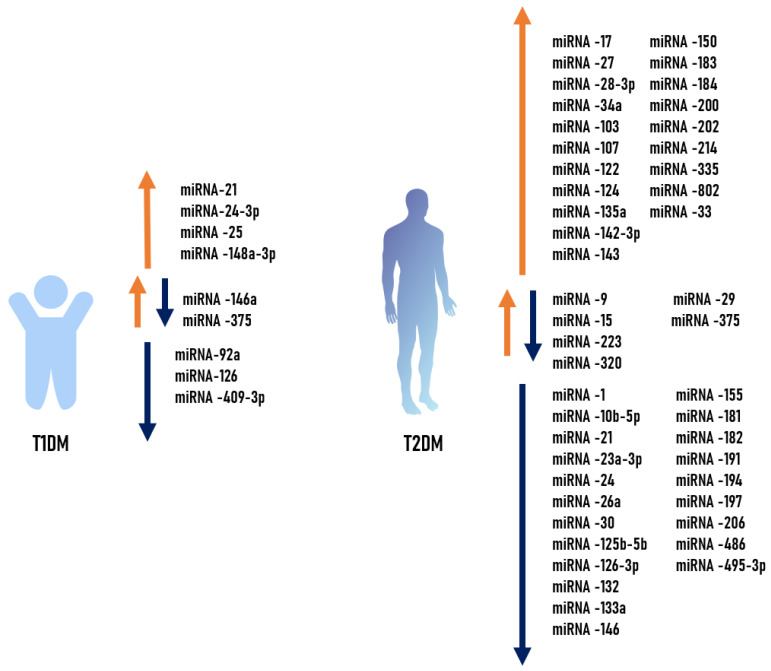
MicroRNAs with altered expression profiles across different types of diabetes. Orange arrows—increase in the level of microRNA expression, blue arrows—decrease in the level of microRNA expression, orange and blue arrows together—increase and decrease in the level of microRNA expression in different sources.

We compared the above-described microRNAs with altered expression profiles across different types of diabetes and attempted to identify overlaps. The results of this analysis are presented in Figure 2.

Currently, there is no reliable biomarker available to assess dysregulation of insulin and glucose homeostasis for diagnosing diabetes prior to its onset. Alterations in the expression profile of certain microRNAs associated with the pathogenesis of T2DM are observed long before the clinical manifestation of the disease and even before prediabetes, which is characterized only by a modest elevation in blood glucose levels. The stage preceding diabetes is reversible and rather indicates a predisposition to diabetes. In this context, microRNAs with the most pronounced expression abnormalities may be further utilized for early diagnosis of diabetes and for predicting disease progression. Aberrant microRNA expression levels, in combination with other clinical information, will enable effective identification of individuals at high risk of developing T2DM and facilitate the timely implementation of preventive measures.

**Figure 3:**
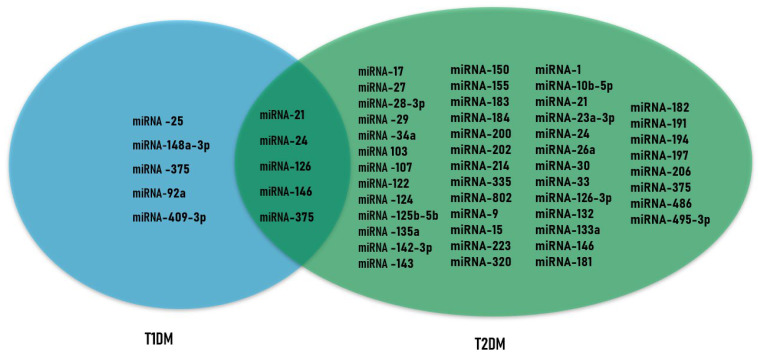
Integration of data on upregulation or downregulation of microRNA expression in different forms of diabetes.

Several microRNAs have been identified as promising for characterizing the state prior to the onset of classic T2DM symptoms. The levels of dysregulated microRNAs may deviate from normal values in either the upward or downward direction (Figure 3), and the period during which these changes occur before the appearance of overt signs of T2DM may range from several months to several years. These are typically circulating microRNAs derived from adipose tissue. Among microRNAs with decreased expression levels, miR-15a [52], miR-19a-3p [38], miR-124a [12], miR-126 [6], and miR-375 [52] can be highlighted. Conversely, increased levels have been observed for miR-28-3p [44], miR-30a-5p [38], miR-103-3p [56], miR-150 [52], and miR-192 [38].

A distinct group of promising biomarkers for prediabetes pathogenesis consists of microRNAs that are directly and/or indirectly involved in the regulation of GLUT4. GLUT4 plays a key role in the process of insulin-induced glucose uptake in muscle and adipose tissues. Aberrant expression of this glucose transporter protein indicates a high risk of developing T2DM [53]. Regulators of GLUT4 with altered expression profiles during the prediabetic stage include miR-17 [38], miR-27a and miR-27a-3p [38], members of the miR-29 family [50], miR-30d [53], miR-223 [6], and miR-320a [38].

Profiling the levels of microRNAs that are most indicative of the prediabetic state will facilitate timely assessment of the risk of developing T2DM and improve prevention strategies among individuals predisposed to diabetes. All the findings are summarized in Table S1.

However, the clinical implementation of microRNAs as novel biomarkers for the early diagnosis and complications is currently limited by several factors: longitudinal studies are still required, findings need validation in independent cohorts, multicenter verification is necessary, and methodological standardization has not yet been achieved. Until these limitations are addressed, microRNAs should be considered promising but not yet clinically applicable candidates.

## 4. Conclusions

Due to distinct pathophysiological processes and clinical manifestations, diagnostic approaches possess certain specificity for different types of diabetes. Nevertheless, despite the differing etiologies of T1DM, T2DM, and GDM, all these types of diabetes share a common feature: impaired insulin secretion, insulin resistance, and altered glucose homeostasis. The regulation of signaling cascades associated with carbohydrate metabolism is mediated by specific microRNAs, which can be considered as diabetes biomarkers. Monitoring aberrant levels of microRNAs involved in the pathogenesis of diabetes and insulin resistance appears to hold promise not only for diagnosing the disease at early stages but also for the future development of strategies aimed at modulating the expression of these key biomarkers. Unfortunately, despite numerous studies indicating the important prognostic and, prospectively, therapeutic roles of microRNAs, the mechanisms through which they contribute to the pathophysiology of diabetes mellitus remain largely unknown. However, the challenge lies in the fact that the regulatory mechanisms of microRNAs are exceedingly complex; each microRNA potentially targets numerous genes, and multiple microRNAs regulate the same gene. This is precisely why a limited set of microRNAs that could be considered reliable biomarkers for diabetes has not yet been defined. At this stage, any proposed microRNA panel represents only a potential diagnostic tool. Such a panel requires further validation and confirmation from clinicians in prospective clinical studies and does not claim clinical readiness. Nevertheless, the development of a universal microRNA panel, rather than the analysis of individual microRNAs, may facilitate the timely diagnosis of different types of diabetes. Subsequently, following the integration of such an approach into clinical practice, it could serve as a foundation for selecting targets for epigenetic therapy.

## Figures and Tables

**Table 1:** T1DM-associated miRNA with representative biological processes.

Category	Representative Biological Processes	miRNAs
β-cell function and insulin secretion	β-cell dysfunction/apoptosis, insulin secretion, glucose homeostasis	miR-21, miR-24-3p, miR-25, miR-146a, miR-148a-3p, miR-375
Inflammation and autoimmunity	Inflammation, autoimmune signaling, immune dysfunction	miR-21, miR-25, miR-148a-3p, miR-409-3p
Endothelial homeostasis	Endothelial dysfunction, endothelial inflammation	miR-92, miR-126

**Table 2:** T2DM-associated miRNA with representative biological processes.

Category	Representative Biological Processes	miRNAs
Insulin resistance and obesity	Insulin resistance, obesity, reduced insulin responsiveness	let-7, miR-1, miR-103, miR-107, miR-126-3p, miR-142-3p, miR-143, miR-150, miR-17, miR-191, miR-197, miR-202, miR-206, miR-214, miR-223, miR-24, miR-320
β-cell function and insulin secretion	β-cell survival, insulin secretion, β-cell differentiation	let-7, miR-124, miR-146a, miR-148a-3p, miR-15, miR-155, miR-184, miR-200, miR-21, miR-24-3p, miR-29, miR-30, miR-375, miR-486, miR-495-3p, miR-9
Glucose metabolism and homeostasis	Glucose transport, hepatic glucose metabolism, homeostasis	let-7, miR-122, miR-124, miR-133a, miR-181, miR-194, miR-23a-3p, miR-24-3p, miR-30, miR-33, miR-802, miR-9
Lipid metabolism and adipose biology	Adipogenesis, adipocyte biology, lipid metabolism	miR-103, miR-107, miR-10b-5p, miR-122, miR-181, miR-182, miR-183, miR-29, miR-33, miR-335, miR-34a
Inflammation and immune regulation	Inflammation, autoimmune signaling, immune dysfunction	miR-126, miR-126-3p, miR-21, miR-29, miR-335, miR-34a,
Insulin signaling and sensitivity	Insulin signaling pathways and insulin sensitivity regulation	miR-132, miR-135a, miR-146, miR-183, miR-194, miR-23a-3p, miR-26a, miR-27, miR-28-3p, miR-335

## Data Availability

No new data were created or analyzed in this study. Data sharing is not applicable.

## Supplementary Materials

The following supporting information can be downloaded at: https://www.mdpi.com/article/10.3390/biom16050742/s1, Table S1: List of miRNA as proposed diagnostic biomarkers of T1DM and T2DM.

## Abbreviations

The following abbreviations are used in this manuscript.

ADA	American Diabetes Association
BDNF	brain-derived neurotrophic factor
Cav-1	caveolin-1
Cxcl10	C-X-C motif chemokine ligand 10
FOXO1	Forkhead box protein O1
GDM	gestational diabetes mellitus
GLUT4	Glucose Transporter Type 4
HLA	Human leukocyte antigen
HNF4α	hepatocyte nuclear factor 4α
IDF	International Diabetes Federation
miRNA	microRNA
PDK1	phosphoinositide-dependent protein kinase 1
MeSH	medical subject
PDX1	pancreatic and duodenal homeobox-1
PTEN	Phosphatase and Tensin Homolog
RCT	Randomized Controlled Trials
SPARC	secreted protein acidic and rich in cysteine
sTNFR	soluble tumor necrosis factor receptor
T1DM	Diabetes mellitus type 1
T2DM	Diabetes mellitus type 2
TRAF3	TNF receptor-associated factor 3
