# Supplementary material for: Epigenetic Regulation Involving microRNAs in Diabetes
Source: Biomolecules. 2026 May 19;16(5):742. doi: 10.3390/biom16050742 (PMC13204174; doi:10.3390/biom16050742)
Supplement: Supplementary file 1 [file biomolecules-16-00742-s001.zip › biomolecules-4291572-supplementary.pdf]

Table S1. List of miRNA as proposed diagnostic biomarkers of T1DM and T2DM.

N	Type of miR	Propose Function	Diabetes Type	Up/Down Regulation	Source	Model
1	miR-1	insulin resistance	T2DM	↘	skeletal muscle	*in vitro* and *in vivo* studies
2	miR-9	insulin release regulation,<br>hepatic glucose homeostasis	T2DM	↘	pancreatic β-cells	animal model
3	miR-10b-5p	regulation of adipocyte function	T2DM	↘	adipose tissue, skeletal muscle	animal model
4	miR-15	insulin secretion	T2DM	↘	serum	Patients
				↗	adipose tissue	
5	miR-17	insulin resistance	T2DM	↗	muscle tissue	animal
6	miR-21	impedes β-cell development,<br>β-cell apoptosis,<br>autoimmune inflammation	T1DM	↗	Plasma<br>Serum<br>PBMC	animal
			T2DM	↘	Plasma	Patients
7	miR-23a-3p	glucose homeostasis,<br>Insulin<br>sensitivity	T2DM	↘	adipose tissue	animal
8	miR-24	insulin resistance	T2DM	↘	Plasma	Patients
				↘	skeletal muscle	
9	miR-24-3p	glucose homeostasis,<br>β-cell dysfunction	T1DM	↗	Plasma<br>Serum	Patients
10	miR-25	inflammation, autoimmune processes, β-cell defects	T1DM	↗	plasma-derived exosomes, serum	Patients
11	miR-26a	insulin sensitivity regulation	T2DM	↘	liver	animal
12	miR-27	insulin signaling pathway	T2DM	↗	plasma-derived exosomes, serum	Patients
13	miR-28-3p	insulin sensitivity regulation	T2DM	↗	serum	Early Patients
14	miR-29	regulation of β-cell function, insulin secretion and lipid metabolism, inflammation	T2DM	↘	liver	Animal
				↗	adipose tissue, skeletal muscle	Animal
15	miR-30	glucose transport regulation,<br>insulin secretion	T2DM	↘	adipose tissue,<br>Serum,<br>Plasma	Patients
16	miR-33	hepatic lipid homeostasis, glucose metabolism	T2DM	↗	liver	Animal
17	miR-34a	adipocyte function regulation, inflammation	T2DM	↗	Serum, adipose tissue	Patients
18	miR-92a	inflammatory pathway regulation, endothelial homeostasis	T1DM	↘	Serum	Patients
19	miR-103, miR-107	adipogenesis, insulin resistance	T2DM	↗	Serum, adipose tissue	Patients
				↗	liver	Animal
20	miR-122	hepatic lipid homeo-	T2DM	↗	Serum, adipose tis-	Patients

		stasis, glucose metabolism, inflammation			sue	
21	miR-124	Regulation of glucose metabolism, insulin secretion, β-cell differentiation	T2DM	↗	β-cells	cultured pancreatic β-cell line
22	miR-125b-5p	inflammatory signaling	T2DM	↘	Tissues	Animals
23	miR-126	inflammatory pathway regulation, endothelial homeostasis	T1DM	↘	Serum	Patients
24	miR-126-3p	Inflammation, insulin resistance	T2DM	↘	Serum<br>Plasma	Patients
25	miR-132	insulin signaling responses	T2DM	↘	Serum<br>Plasma	Patients, Animals
26	miR-133a	cardiovascular complications,<br>glucose uptake regulation	T2DM	↘	Serum<br>Plasma<br>Skeletal muscle	Patients
27	miR-135a	reduces insulin signaling	T2DM	↗	skeletal muscle	Patients
28	miR-142-3p	insulin resistance, Obesity	T2DM	↗	Plasma	Patients
29	miR-143	insulin resistance, Obesity	T2DM	↗	Plasma	Patients
					Liver	Animals
30	miR-146	inflammatory signaling, hepatic glycogenesis, insulin signaling	T2DM	↘	Plasma<br>Serum	Patients
31	miR-146a	β-cell function and survival	T1DM	↗	Plasma	Patients
				↘	PBMC	
32	miR-148a-3p	Inflammation regulation, β-cell apoptosis	T1DM	↗	Plasma<br>Serum<br>T-cells<br>PBMC	Patients
33	miR-150	insulin resistance, Obesity	T2DM	↗	Plasma	Patients
34	miR-155	β-cell function maintaining	T2DM	↘	Serum	Patients
35	miR-181	adipocyte function regulation, glucose homeostasis, insulin sensitivity	T2DM	↘	Serum	Patients
					adipose tissue	Animals
36	miR-182	adipocyte function regulation	T2DM	↘	skeletal muscle, adipose tissue	Patients
37	miR-183	Adipogenesis, insulin signaling	T2DM	↗	hepatocytes	Patients
38	miR-184	β-cells protection	T2DM	↗	adipose tissue	Patients
39	miR-191	insulin resistance, Obesity	T2DM	↘	Plasma	Patients
40	miR-194	insulin signaling, glucose metabolism	T2DM	↘	skeletal muscle	Patients
41	miR-197	insulin resistance,	T2DM	↘	Plasma	Patients

		Obesity				
42	miR-200	Insulin secretion	T2DM	↗	β-cells	cultured pancreatic β-cell line
43	miR-202, miR-214	insulin resistance regulation	T2DM	↗	skeletal muscle	Patients
44	miR-206	insulin resistance regulation	T2DM	↘	skeletal muscle	Patients
45	miR-223	insulin resistance regulation	T2DM	↘	Serum	Patients
				↗	adipose tissue	
46	miR-320	insulin resistance regulation	T2DM	↘	Serum	Patients
47	miR-335	Inflammation, insulin signaling, lipid metabolism	T2DM	↗	Plasma Serum PBMC	Patients
48	miR-375	insulin secretion modulating	T1DM	↗	Plasma Serum PBMC	Patients
		insulin secretion regulation, β-cell development, glucose homeostasis	T2DM	↗	Pancreas	Animals
				↘	Plasma Serum adipose tissue	Patients
49	miR-409-3p	autoimmune inflammation	T1DM	↘	Plasma	Newly Patients
50	miR-486	β-cells function	T2DM	↘	Plasma	Patients
51	miR-495-3p	β-cells function	T2DM	↘	adipose tissue	Patients
52	miR-802	hepatic glucose production Inhibition	T2DM	↗	hepatocytes	Animal
53	let-7	insulin secretion, insulin resistance, glucose tolerance	T2DM	↗	adipose tissue, Plasma	Patients
				↗	Pancreas, skeletal muscle	Animal
				↘	hepatocytes	

PBMC—peripheral blood mononuclear cells.
